# COVID-19 IgG/IgM patterns, early IL-6 elevation and long-term radiological sequelae in 75 patients hospitalized due to interstitial pneumonia followed up from 3 to 12 months

**DOI:** 10.1371/journal.pone.0262911

**Published:** 2022-02-22

**Authors:** Domenico Maurizio Toraldo, Francesco Satriano, Rodolfo Rollo, Gabriella Verdastro, Giovanni Imbriani, Emanuele Rizzo, Alberto Argentiero, Andrea Falco, Paolo Ambrosino, Alessandro Miani, Prisco Piscitelli

**Affiliations:** 1 Department of Rehabilitation, Cardiorespiratory Rehabilitation Unit–“V. Fazzi Hospital”, Local Health Authority ASL Le, Lecce, Italy–Lecce, Italy; 2 Covid Sub-intensive Respiratory Unit–“V. Fazzi Hospital”, Local Health Authority ASL Le, Lecce, Italy–Lecce, Italy; 3 Local Health Authority, ASL Le, Lecce, Italy; 4 University of Salento, Lecce, Italy; 5 University of Parma, Parma, Italy; 6 Department of Sciences and Technology, University of Sannio, Benevento, Italy; 7 Department of Environmental Science and Policy, University of Milan, Milan, Italy; 8 Italian Society of Environmental Medicine (SIMA), Milan, Italy; Kaohsuing Medical University Hospital, TAIWAN

## Abstract

**Background:**

COVID-19 pandemic resulted in about 165 million infections and 3.4 million deaths all over the world across 15 months. The most severe clinical presentation of COVID-19 diseases is interstitial pneumonia.

**Methods:**

In this paper we describe clinical outcomes based on radiological features as well as the pattern of haematochemical parameters and IgG/IgM antibodies in 75 patients hospitalized due to COVID-related interstitial pneumonia not requiring intensive care assistance. Each patient underwent routine laboratory tests, including inflammatory markers and coagulation profile at baseline. Computed Tomography (CT) was performed at baseline and after 3 months to assess the persistence of radiological sequelae. A Generalized Linear Model (GLM) was used to test for each patient the association between individual haematochemical parameters at the time of hospital admission and the subsequent radiological features after three months. The presence of IgG antibodies was quantitatively determined in 70 patients at the time of hospital admission and after 3 months. A subgroup of 49 and 21 patients underwent additional dosage of IgG after 6 and 12 months, respectively. IgM serological antibodies were available for 17 patients at baseline and 61 at T3, with additional follow-up for 51 and 20 subjects after 6 and 12 months, respectively.

**Results:**

Only 28 out of 75 patients discharged from the hospital were totally healed after 3 months, while 47 patients (62.7%) still presented radiological sequelae. According to the GLM model, specific haematochemical baseline parameters—such as IL-6, GPT, platelets and eosinophil count—showed a statistically significant association with the presence of radiological sequelae at month 3 highlighting an OR = 0.5, thus meaning that subjects completely healed after 3 months presented half levels of IL-6 at baseline compared to patients with sequelae. In general, IgG serum levels were always higher than IgM at the time of hospitalization (75% at T0; n = 12 out of 16 patients with data available in both visits), after 3 months (72.1%; n = 44 out of 61 pts.), after 6 months (56.8%; 25 out of 44 pts.), and one year after hospitalization (60%; 12 out of 20 pts.). Overall, IgG and IgM serum levels presented a statistically significant decreasing trend from the baseline to month 3, 6 and 12. One patient presented an increase in IgM between baseline and month 3 but negative PCR test for SARS-COV2 on throat swab.

**Conclusions:**

As supported by our findings on 75 patients, COVID-related interstitial pneumonia triggers early IgG levels (higher than IgM) that gradually decrease over 12 months. Mid-term sequelae are still detectable at lung Computed Tomography after 3 months from the hospital admission. Occasionally, it is possible to observe increase of IgM levels in presence of low concentrations of IgG and negative PCR ELISA tests for SARS-COV2 RNA. Baseline levels of IL-6 could be proposed as predictor of radiological mid/long-term sequelae after COVID-related interstitial pneumonia.

## Introduction

Severe Acute Respiratory Syndrome Coronavirus 2 (SARS-CoV-2) is responsible for coronavirus disease-19 (COVID-19), resulted in about 165 million infections and 3.4 million deaths all over the world. Increasing evidence in humans suggest that the virus is responsible for the production of IgA, IgM and IgG neutralizing antibodies against viral nucleocapside (N) and spike (S) proteins after the infection [1]. Interestingly, the vast majority of them are asymptomatic or mild-symptomatic, with no need for medical assistance.

Indeed, the high number of infected individuals suggests that they can contribute to the spread of the novel coronavirus among their communities and should therefore be included in the assessment of infection risk [2]. However, the lifespan of neutralizing antibodies is still matter of debate. Based on literature data, the latter depends on different factors. The length of the infection and severity of the disease are the most relevant from a clinical point of view [3]. Indeed, recent studies [4–6], demonstrated a rapid decline of antibodies post the onset of symptoms (POS), even though some individuals showed high levels of neutralizing antibodies after 60 days POS [7–9]. Moreover, the conversion of Ig still needs to be clarified. Initial studies suggest that the IgG can be produced earlier or simultaneously compared to IgM or IgA [10–12]. However, it has been reported that few days POS some individuals only displayed neutralizing IgM or IgA activity, thus suggesting that the capacity of these latter antibodies could be involved in virus neutralization even in absence of IgG [7, 13]. The production of neutralizing IgA at early stages of the infection could effectively eliminate the virus in the respiratory mucosa, whereas the persistence of both IgA and IgG in the serum over the time can trigger the immune response in mild cases [13].

Notably, the high levels of IgG in severe cases may trigger both the innate and adaptive immunity to efficiently eliminate the novel coronavirus [14, 15]. It has been observed that the kinetics of SARS-CoV-2 IgM antibodies reaches a peak after 14 days POS, while IgG levels persist over time until a rapid decline 5–6 months after the onset of the disease [9, 16, 17]. At the same time, the possibility of asymptomatic re-infection has been reported [18]. There are also increasing evidence of possible predictors of disease severity such as IL-6 [19, 20], which has also been identified as a potential target for pharmaceutical treatments [21–23]. In this paper we describe the kinetics and patterns of IgG/IgM antibodies, clinical outcomes based on radiological features and baseline concentration of specific haematochemical parameters measured in 75 patients hospitalized due to COVID-related interstitial pneumonia (with mild to severe symptoms not requiring Intensive Care Unit assistance) followed for 3, 6 and 12 months.

## Methods

We analyzed IgG and IgM neutralizing antibodies in 74 patients with PCR-confirmed positive tests to SARS-COV-2 RNA (Polymerase Chain Reaction performed with device DxC 700 AU Chemistry Analyzer Beckman Coulter, Indianapolis USA) on a total of 75 subjects hospitalized at the Division of Pneumology of San Cesario COVID Hospital (Local Health Authority ASL Lecce) between April and September 2020 with diagnosis of COVID19-related interstitial pneumonia confirmed by computed tomography (CT) presenting mild to severe symptoms. All hospitalized patients were treated according to the currently available international/national guidelines or protocols in the frame of Good Clinical Practice and required oxygen administration (all day long) at the moment of hospital admission, but not Intensive Care Unit assistance.

The presence of IgG anti-nucleocapside (N) antibodies was quantitatively determined (with analyzer DiaSorin Liaison XL, Vercelli, Italy) for 69 patients at the time of hospital admission and in 70 patients after 3 months during the first follow-up visit. A subgroup of 49 patients underwent a second dosage of IgG after six months and 21 of them had a further IgG determination at 12 months. In a subgroup of 17 patients we also determined IgM levels anti-spike protein (S) at T0 (with analyzer Alinity, Abbott Diagnostics, Chicago, USA), but at month 3 (T3) we had IgM data available for 61 patients. IgM levels were also available for 51 and 20 subjects at 6 months (T6) and after one year (T12), respectively. The significance in the changes of IgG and IgM antibodies between the baseline and 3-6-12 months was assessed by repeated measure ANOVA by using F-test to statistically test the equality of means.

Computed Tomography (CT) examination was performed (Brillance TC Philips 16 Slice, model 2011) for all patients along with pneumological visit at baseline and after 3 months while undergoing the first follow up visit in order to assess the persistence of radiological sequelae affecting physiological lung function. Radiological classification was based on the findings officially certified by the radiologists who performed the Computed Tomography. The presence of the following radiological findings at CT examination was indicating mild post-pneumonia features: leucocitaries infiltrates, lymphadenomegaly, lymphadenopathy, generic alterations of lung radiological aspect, pervulosity lymph node, broncovascular texture reinforcement. The following radiological findings at CT examination were classified as indicative of severe post-pneumonia features: infiltrative lesions, areas of parenchymal hyperdensity (framed glass), pleural effusion, pericardial effusion, focal nodular lesions, lesions or thickening and parenchymal consolidation phenomena with an infiltrative / expansive character, micronodular and nodular formations or microformations, pleural thickening or consolidation areas, dense or lamellar peri-lesional striae at the bases, dense or lamellar striae of fibrotic or ectatic type, fibro-sclerotic aspects at the apices, sub-pleural interstitium thickening, pleural thickening, irregularity with frayed appearance, para-fibrotic emphysema, sub-pleural fibrosclerotic aspects, thickening of pericardial sheets, fibro-cicatricial striae, inter or intralobular interstitium thickening, interstitial septa thickening, disventilative diaphragmatic features, intra-scissural effusion, paraseptal and bullous aspects of fibrotic-retracting emphysema, scissural thickening, basal interstitial thickening, peri-broncovascular interstitium thickening, ectasia of bronchial and bronchiolar structures, cicatricial aspects, bullous-cystic formations or microformations.

At the time of hospital admission, each patient underwent routine laboratory tests on blood serum samples to determine the following parameters: RBC (x 10^6/μL), WBC (x 10^3/μL), HGB (g/dL), HCT (%), MCV (fL), MCH (pg), MCHC (g/dL), PLT(x 10^3/μL), RDW-SD (fL), RDW-CV (%), PDW (fL), MPV (fL), P-LCR (%), NEUT%, LINF%, BAS%, EOS%, MONOC%, VES (mm/h), PT (%), INR, PTT (s), FIBRINOGEN (mg/dL), D-DIMER (ng/mL), AZOTEMIA (mg/dL),CREATININ (mg/dL), Egfr, GOT (U/L), GPT (U/L),GGT (U/L),LDH (U/L), CPK (U/L), Bilirubin total/direct/indirect (mg/dl), PCR (mg/dl), VES (mg/dl), IL-6 (pg/mL), FERRITIN (ng/mL), PROCALCITONIN (ng/mL), NT-proBNP (pcg/mL), TROPONIN HS (ng/L), OMOCISTEIN (mcmol/L). We used a Generalized Linear Model (GLM) considering the baseline haematochemical exams as dependent variables and the 3-month clinical outcome (healing or long-term sequelae based on radiological evidence of healing) as independent variable. Only the dependent variables which resulted significantly associated with the independent ones (clinical outcome) were kept and run in the model. The model was implemented with a method of stepwise selection on dependent variables (full model). The selections were analyzed considering the test AIC for each step. Both the correlation coefficient and the VIF (Variation Inflation Factor) were used to verify multicollinearity: the ratio between VIF and R^2^ is inversely proportional and is equal to 1/1-R^2^, while multicollinearity is certain at a level of 0.9 of correlation coefficient (or higher), and the VIF values for the included variables should be less than 10. The proposed model is the best performing in terms of AIC (Akaike Information Criterion) as it also guarantees the lowest level of collinearity among the variables. The calculation of VIF (by a value of R^2^ = 0.5) in our model presented a value of 2.0, far away from the critical VIF value set at 10.0 for showing multicollinearity.

## Results

**Fig 1:**
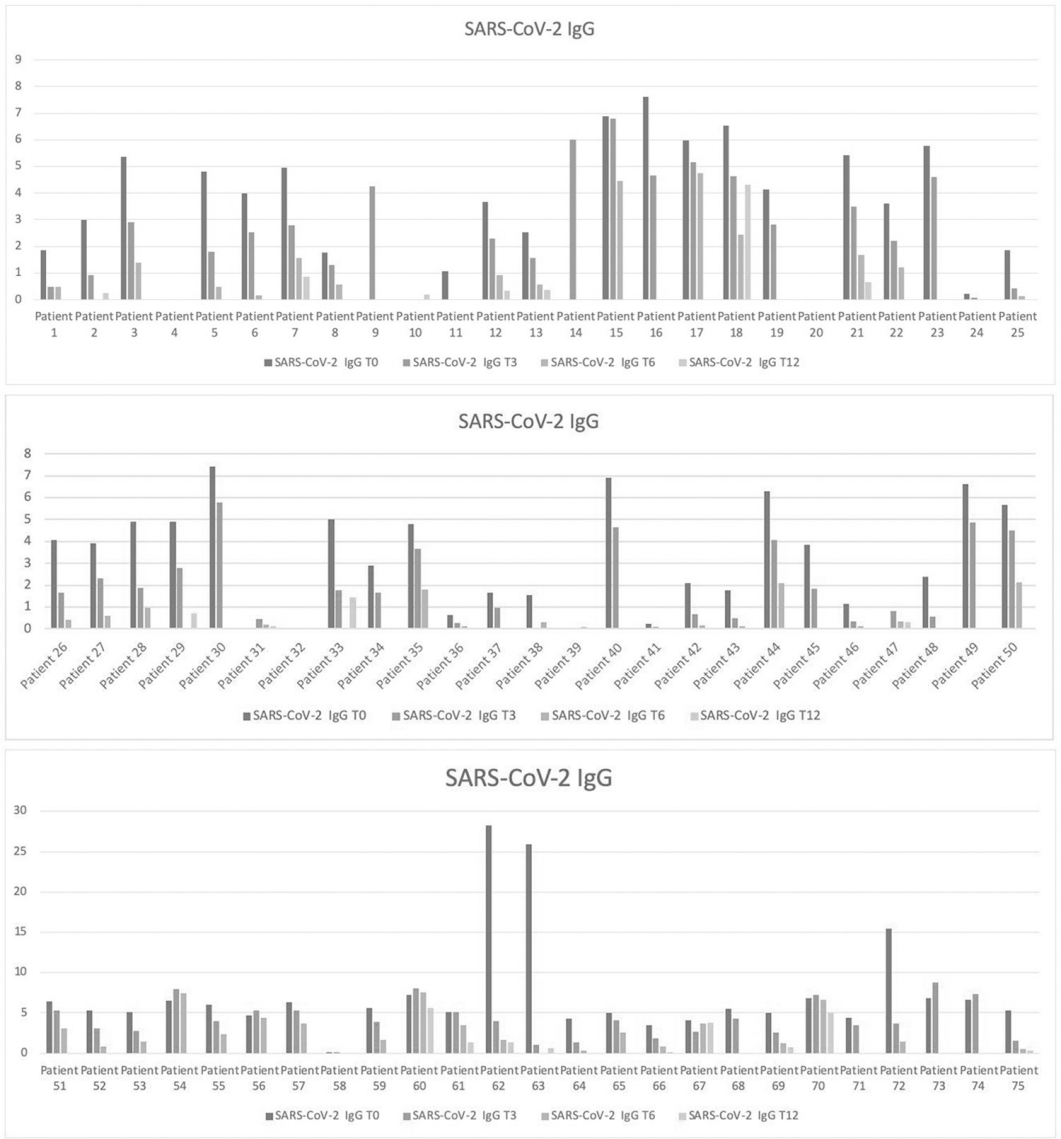
**a.** Serum IgG antibodies levels in a subgroup of patients from 1 to 25. Serum IgG antibodies levels in a subgroup of patients from 1 to 25 at baseline (T0; blue line), month 3 (T3; orange line), month 6 (T6; gray line and month 12 (T12 Yellow line data available on a smaller subgroup of 7 patients). **b.** Serum IgG antibodies levels in a subgroup of patients from 26 to 50. Serum IgG antibodies levels in a subgroup of patients from 26 to 50 at baseline (T0; blue line), month 3 (T3; orange line), month 6 (T6; gray line and month 12 (T12 Yellow line data available on a smaller subgroup of 5 patients). **c.** Serum IgG antibodies levels in a subgroup of patients from 51 to 75. Serum IgG antibodies levels in a subgroup of patients from 51 to 75 at baseline (T0; blue line), month 3 (T3; orange line), month 6 (T6; gray line and month 12 (T12 Yellow line data available on a smaller subgroup of 9 patients).

**Fig 2:**
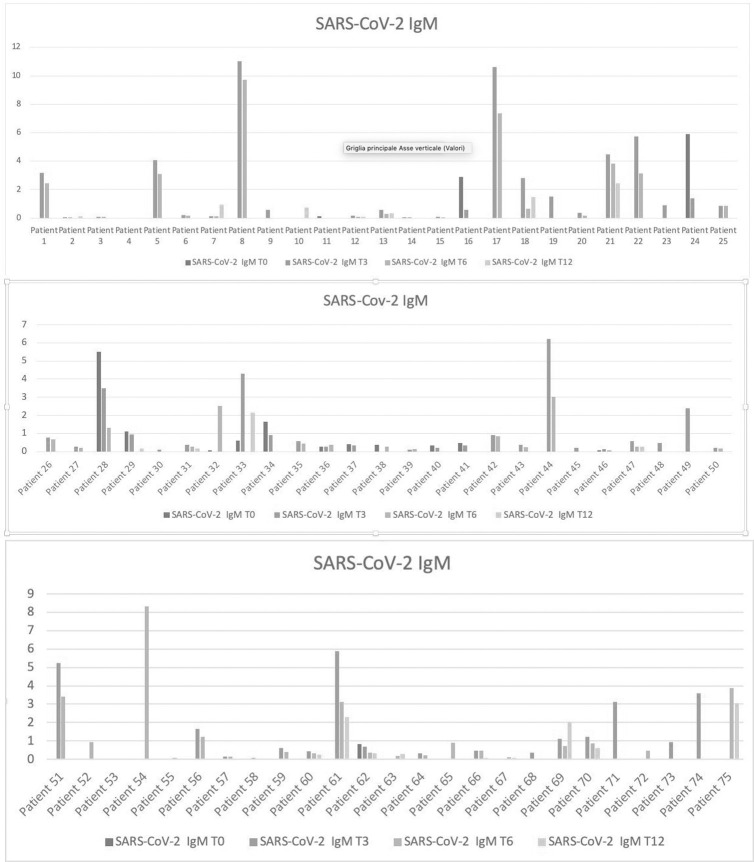
**a.** Serum IgM Antibodies levels in a subgroup of patients from 1 to 25. Serum IgM Antibodies levels in a subgroup of patients from 1 to 25 at baseline (T0; blue line), month 3 (T3; orange line), month 6 (T6; grey line), month 12 (T12; yellow line). **b.** Serum IgM Antibodies levels in a subgroup of patients from 26 to 50. Serum IgM Antibodies levels in a subgroup of patients from 26 to 50 at baseline (T0; blue line), month 3 (T3; orange line), month 6 (T6; grey line), month 12 (T12; yellow line). **c.** Serum IgM Antibodies levels in a subgroup of patients from 51 to 75. Serum IgM Antibodies levels in a subgroup of patients from 51 to 75 at baseline (T0; blue line), month 3 (T3; orange line), month 6 (T6; grey line), month 12 (T12; yellow line).

As showed in Table 1 and resumed in Fig 1A–1C, data concerning IgG were available for a total of 69 patients at the baseline (T0), 70 patients after three months (T3), 49 at month 6 (T6) and 21 after twelve months (T12). IgM serological antibodies level were available for 17 patients at baseline, 61 at T3, 51 at T6 and 20 subjects at T12 (Fig 2A–2C).

**Table 1:** Serum levels of IgG and IgM antibodies determined at baseline (T0), month 3 (T3), month 6 (T6) and month 12 (T12).

	SARS-CoV-2 IgG (index value)				SARS-CoV-2 IgM (index value)			
** **	**T0**	**T3**	**T6**	**T12**	**T0**	**T3**	**T6**	**T12**
**Patient 1**	**1,87**	**0,48**	**0,48**	** NA**	**NA**	**3,18**	**2,43**	**NA**
**Patient 2**	**2,99**	**0,91**	**NA**	**0,26**	**0,06**	**0,05**	**NA**	**0,11**
**Patient 3**	**5,37**	**2,91**	**1,38**	**NA**	**NA**	**0,1**	**0,1**	**NA**
**Patient 4**	** NA**	** NA**	** NA**	** NA**	** NA**	** NA**	** NA**	** NA**
**Patient 5**	**4,8**	**1,79**	**0,48**	** NA**	** NA**	**4,09**	**3,11**	** NA**
**Patient 6**	**4**	**2,53**	**0,16**	** NA**	** NA**	**0,21**	**0,16**	** NA**
**Patient 7**	**4,95**	**2,79**	**1,58**	**0,85**	** NA**	**0,11**	**0,12**	**0,93**
**Patient 8**	**1,77**	**1,29**	**0,58**	** NA**	** NA**	**11,02**	**9,73**	**NA**
**Patient 9**	** NA**	**4,24**	** NA**	** NA**	** NA**	**0,59**	** NA**	** NA**
**Patient 10**	** NA**	** NA**	** NA**	**0,2**	** NA**	** NA**	** NA**	**0,72**
**Patient 11**	**1,07**	** NA**	** NA**	**NA**	**0,11**	** NA**	** NA**	**NA**
**Patient 12**	**3,66**	**2,29**	**0,93**	**0,34**	**NA**	**0,18**	**0,1**	**0,09**
**Patient 13**	**2,53**	**1,58**	**0,58**	**0,38**	**NA**	**0,56**	**0,29**	**0,32**
**Patient 14**	**NA**	**6,01**	**NA**	** NA**	**0,04**	**0,04**	**NA**	** NA**
**Patient 15**	**6,88**	**6,79**	**4,46**	** NA**	**NA**	**0,07**	**0,06**	** NA**
**Patient 16**	**7,62**	**4,66**	**NA**	** NA**	**2,89**	**0,59**	**NA**	** NA**
**Patient 17**	**5,98**	**5,15**	**4,75**	** NA**	** NA**	**10,59**	**7,36**	** NA**
**Patient 18**	**6,53**	**4,64**	**2,43**	**4,31**	** NA**	**2,82**	**0,65**	**1,46**
**Patient 19**	**4,14**	**2,82**	**NA**	** NA**	** NA**	**1,51**	**NA**	**NA**
**Patient 20**	**0,03**	**0,01**	**0,01**	** NA**	** NA**	**0,37**	**0,18**	**NA**
**Patient 21**	**5,42**	**3,5**	**1,67**	**0,66**	** NA**	**4,46**	**3,83**	**2,45**
**Patient 22**	**3,61**	**2,22**	**1,22**	** NA**	** NA**	**5,75**	**3,15**	** NA**
**Patient 23**	**5,78**	**4,6**	** NA**	** NA**	** NA**	**0,91**	** NA**	** NA**
**Patient 24**	**0,22**	**0,07**	** NA**	** NA**	**5,91**	**1,38**	** NA**	** NA**
**Patient 25**	**1,87**	**0,44**	**0,13**	** NA**	** NA**	**0,88**	**0,87**	** NA**
**Patient 26**	**4,05**	**1,66**	**0,4**	** NA**	** NA**	**0,77**	**0,66**	** NA**
**Patient 27**	**3,93**	**2,3**	**0,58**	** NA**	** NA**	**0,26**	**0,22**	** NA**
**Patient 28**	**4,9**	**1,87**	**0,96**	** NA**	**5,52**	**3,5**	**1,3**	** NA**
**Patient 29**	**4,89**	**2,78**	** NA**	**0,71**	**1,1**	**0,96**	** NA**	**0,18**
**Patient 30**	**7,42**	**5,79**	** NA**	** NA**	** NA**	**0,11**	** NA**	**NA**
**Patient 31**	**NA**	**0,46**	**0,21**	**0,11**	**NA**	**0,36**	**0,27**	**0,17**
**Patient 32**	**0,01**	**NA**	**0,01**	** NA**	**0,06**	**NA**	**2,53**	** NA**
**Patient 33**	**5,03**	**1,77**	** NA**	** 1,42**	**0,61**	**4,31**	** NA**	**2,16**
**Patient 34**	**2,89**	**1,66**	** NA**	** NA**	**1,64**	**0,91**	** NA**	** NA**
**Patient 35**	**4,81**	**3,66**	**1,79**	** NA**	**NA**	**0,56**	**0,44**	** NA**
**Patient 36**	**0,64**	**0,27**	**0,14**	** NA**	**0,28**	**0,27**	**0,36**	** NA**
**Patient 37**	**1,66**	**0,95**	**NA**	** NA**	**0,42**	**0,34**	**NA**	** NA**
**Patient 38**	**1,54**	**NA**	**0,3**	** NA**	**0,36**	**NA**	**0,27**	** NA**
**Patient 39**	**0,05**	**0,06**	**0,08**	** NA**	**NA**	**0,09**	**0,14**	** NA**
**Patient 40**	**6,91**	**4,65**	** NA**	** NA**	**0,34**	**0,2**	**0**	** NA**
**Patient 41**	**0,23**	**0,09**	** NA**	** NA**	**0,48**	**0,33**	**NA**	** NA**
**Patient 42**	**2,08**	**0,66**	**0,17**	** NA**	** NA**	**0,9**	**0,85**	** NA**
**Patient 43**	**1,76**	**0,48**	**0,13**	** NA**	** NA**	**0,36**	**0,25**	** NA**
**Patient 44**	**6,3**	**4,07**	**2,08**	** NA**	** NA**	**6,22**	**3,03**	** NA**
**Patient 45**	**3,84**	**1,85**	**NA**	** NA**	** NA**	**0,22**	**NA**	** NA**
**Patient 46**	**1,14**	**0,34**	**0,11**	**0,05**	**0,07**	**0,15**	**0,07**	** NA**
**Patient 47**	**NA**	**0,81**	**0,36**	**0,29**	**NA**	**0,58**	**0,26**	**0,26**
**Patient 48**	**2,37**	**0,55**	** NA**	** NA**	** NA**	**0,48**	** NA**	** NA**
**Patient 49**	**6,62**	**4,86**	** NA**	** NA**	** NA**	**2,4**	** NA**	** NA**
**Patient 50**	**5,68**	**4,49**	**2,14**	** NA**	** NA**	**0,2**	**0,17**	** NA**
**Patient 51**	**6,4**	**5,29**	**3,03**	** NA**	** NA**	**5,22**	**3,42**	** NA**
**Patient 52**	**5,25**	**3,03**	**0,8**	** NA**	** NA**	** NA**	**0,93**	** NA**
**Patient 53**	**5,06**	**2,75**	**1,42**	** NA**	** NA**	** NA**	**0,05**	** NA**
**Patient 54**	**6,47**	**7,9**	**7,44**	** NA**	** NA**	** NA**	**8,33**	** NA**
**Patient 55**	**6,05**	**4,03**	**2,32**	** NA**	** NA**	** NA**	**0,07**	** NA**
**Patient 56**	**4,72**	**5,35**	**4,39**	** NA**	** NA**	**1,67**	**1,22**	** NA**
**Patient 57**	**6,31**	**5,31**	**3,71**	** NA**	** NA**	**0,16**	**0,15**	** NA**
**Patient 58**	**0,13**	**0,1**	**NA**	** NA**	** NA**	**0,09**	**NA**	** NA**
**Patient 59**	**5,61**	**3,92**	**1,65**	** NA**	** NA**	**0,62**	**0,4**	** NA**
**Patient 60**	**7,2**	**8,09**	**7,58**	**5,62**	** NA**	**0,43**	**0,32**	**0,25**
**Patient 61**	**5,12**	**5,05**	**3,46**	**1,31**	** NA**	**5,89**	**3,12**	**2,29**
**Patient 62**	**28,25**	**3,96**	**1,63**	**1,37**	**0,83**	**0,69**	**0,37**	**0,31**
**Patient 63**	**25,96**	**1**	**NA**	**0,64**	** NA**	**NA**	**0,18**	**0,3**
**Patient 64**	**4,26**	**1,36**	**0,33**	** NA**	** NA**	**0,31**	**0,23**	** NA**
**Patient 65**	**4,95**	**4,06**	**2,6**	** NA**	** NA**	**NA**	**0,9**	** NA**
**Patient 66**	**3,52**	**1,8**	**0,83**	**0,11**	** NA**	**0,48**	**0,47**	**0,08**
**Patient 67**	**4,06**	**2,69**	**3,66**	**3,77**	** NA**	**NA**	**0,1**	**0,06**
**Patient 68**	**5,53**	**4,25**	**NA**	**NA**	** NA**	**0,36**	** NA**	** NA**
**Patient 69**	**4,95**	**2,55**	**1,22**	**0,71**	** NA**	**1,1**	**0,73**	**2**
**Patient 70**	**6,79**	**7,28**	**6,63**	**4,96**	** NA**	**1,23**	**0,88**	**0,61**
**Patient 71**	**4,42**	**3,48**	**NA**	** NA**	** NA**	**3,12**	** NA**	** NA**
**Patient 72**	**15,43**	**3,69**	**1,4**	** NA**	** NA**	**NA**	**0,48**	** NA**
**Patient 73**	**6,83**	**8,79**	** NA**	** NA**	** NA**	**0,94**	** NA**	** NA**
**Patient 74**	**6,67**	**7,33**	** NA**	** NA**	** NA**	**3,6**	** NA**	** NA**
**Patient 75**	**5,3**	**1,5**	**0,5**	**0,33**	** NA**	**NA**	**3,89**	**3,06**

*NA: Not Available

### Comparison of IgG and IgM serum levels from 0 to 12 months

We had available both IgG and IgM values at the baseline (T0) only for 16 patients with 12 of them (75%) presenting IgG values higher than IgM. After three months (T3), information about both IgG and IgM levels were available for a total of 61 patients; among them, 44 subjects (72%) had higher IgG values than IgM, 1 person had equal values and the remaining 16 (26%) presented lower values of IgG compared to IgM. At month six (T6), IgG and IgM values were simultaneously available for 44 patients, with 25 of them (56.8%) presenting IgG values higher than IgM, 1 had equal serum levels of both kind of antibodies while the remaining 18 (40.9%) presented lower values of IgG compared to those of IgM. After 12 months (T12), we had available both IgG and IgM serum levels only for 20 patients, with 12 of them (60%) presenting higher IgG values than IgM.

### IgG serum levels from 0 to 12 months

IgG values at the baseline (T0) and after three months (T3) were simultaneously available for 66 patients (with 58 of them, namely 87.8%, presenting higher values of IgG at the baseline compared to T3), but for 44 of these subjects we had also available IgG concentrations at T6, that showed a constant decrease in 41 subjects compared to T0. Only for 14 patients, serum levels of IgG were available up to T12 showing always lower values after one year compared to the baseline, although some fluctuations between month 3 and 6 were detectable in 4 patients. Overall, IgG serum levels showed a statistically significant reduction from T0 to T12 (p<0,001), with a lower effect detectable at one year compared to intermediate follow-up visits (T3 and T6) due to a small number of patients observed at T12.

### IgM serum levels from 0 to 12 months

IgM values at the baseline (T0) and after three months (T3) were available for 14 patients: 11 of them (78.5%) had higher IgM values at the baseline than at T3 and 1 had equal values. A female patient showed an increase of IgM between baseline and month 3 (higher than IgG levels), despite a double negative result for SARS-COV2 RNA (ELISA test on two consecutive throat swabs). Extended data about serum levels of IgM from T0 up to T3 and T6 were available for 4 patients (showing always a decrease from T0 to T6), while only one of these subjects underwent additional serum determination of IgM also after 12 months (showing a stable decrease across one year). However, a larger number (n = 38) of serum IgM levels were available between T3 and T6 (lacking of T0 determinations), showing a general decreasing trend in 34 patients (89,4%), stable values in 1 case, and an increase in 3 subjects. Twelve of these 38 subjects were followed up for IgM concentrations from T3 up to one year, presenting always constant reductions between month 3 and 12 in all the patient except in two (with some fluctuations between T3 and T6 in four patients). Overall, IgM serum levels showed a statistically significant reduction from T0 to T12 (p<0,001), with a lower effect detectable at one year compared to intermediate follow-up visits (T3 and T6) due to a small number of patients observed at T12.

### Radiological features and haematochemical parameters

Table 2 describes the specific findings observed at Computed Tomography at baseline and after 3 months per each of the 75 hospitalized patients.

**Table 2:** Specific findings observed at Computed Tomography at baseline (T0) and after 3 months (T3) per each of the 75 hospitalized patients.

	*Infiltrative lesions or parenchymal hyperdensity with framed glass aspect*		*Lymphadenomegalies*, *Lymphadenopathies or nodularities*		*Pleural or pericardial thickness/effusion*		*Lung plot thickness/ectasia or fibrotic aspects*		*Alterations*, *lesions or thickening of parenchimal or pleural base*	
	T0	T3	T0	T3	T0	T3	T0	T3	T0	T3
PATIENT 1	0	NA	0	1	0	0		0		
PATIENT 2	1	NA	0	NA	0	NA	NA	NA	1	NA
PATIENT 3	2	0	NA	0	NA	0	2	2	2	2
PATIENT 4	1	2	1	1	0	1	2	NA	1	1
PATIENT 5	NA	NA	1	NA	0	NA	0	NA	NA	NA
PATIENT 6	1	2	1	1	2	1	2	2	1	1
PATIENT 7	NA	0	NA	0	0	1	1	NA	1	1
PATIENT 8	0	0	1	1	0	0	0	0	1	1
PATIENT 9	1	NA	1	0	0	0	NA	NA	NA	NA
PATIENT 10	0	NA	1	NA	0	NA	NA	NA	1	NA
PATIENT 11	1	NA	1	NA	1	NA	NA	NA	1	NA
PATIENT 12	2	2	1	2	2	2	2	2	NA	2
PATIENT 13	2	0	1	1	0	0	2	NA	NA	NA
PATIENT 14	2	2	1	0	NA	0	NA	NA	1	1
PATIENT 15	2	NA	1	1	0	0	2	2	1	2
PATIENT 16	1	NA	1	NA	0	NA	2	NA	NA	NA
PATIENT 17	1	2	0	0	0	0	1	0	NA	1
PATIENT 18	1	1	1	1	0	1	1	1	1	NA
PATIENT 19	1	2	1	1	0	1	1	NA	1	1
PATIENT 20	1	2	1	1	0	0	1	2	1	2
PATIENT 21	0	0	0	0	0	0	0	0	NA	
PATIENT 22	1	0	1	1	0	0	1	0	NA	1
PATIENT 23	NA	NA	1	1	0	0	1	1	NA	NA
PATIENT 24	0	NA	0	NA	0	NA	0	NA	0	NA
PATIENT 25	0	NA	1	NA	0	NA	0	NA	NA	NA
PATIENT 26	0	0	1	1	NA	0	0	0	NA	NA
PATIENT 27	0	0	0	0	0	0	0	0	NA	NA
PATIENT 28	0	NA	0	NA	0	NA	1	NA	NA	NA
PATIENT 29	0	NA	1	NA	0	NA	NA	NA	1	NA
PATIENT 30	1	1	1	1	1	0	NA	1	1	1
PATIENT 31	0	0	0	0	0	0	0	0	1	1
PATIENT 32	0	NA	0	NA	0	NA	0	NA	1	NA
PATIENT 33	NA	NA	NA	NA	NA	NA	NA	NA	NA	NA
PATIENT 34	1	NA	0	NA	0	NA	0	NA	NA	NA
PATIENT 35	1	2	1	1	0	0	1	1	1	1
PATIENT 36	1	NA	1	NA	0	NA	NA	NA	1	NA
PATIENT 37	0	NA	1	1	0	NA	0	NA	NA	NA
PATIENT 38	0	NA	0	NA	0	NA	0	NA	1	NA
PATIENT 39	1	1	1	1	0	0	NA	NA	1	NA
PATIENT 40	0	NA	1	NA	0	NA	NA	NA	1	NA
PATIENT 41	0	NA	1	NA	1	NA	1	NA	1	NA
PATIENT 42	0	NA	0	NA	0	NA	0	NA	NA	NA
PATIENT 43	1	1	0	1	0	0	NA	NA	1	1
PATIENT 44	1	NA	1	NA	0	NA	NA	NA	NA	NA
PATIENT 45	NA	NA	1	NA	1	NA	1	NA	1	NA
PATIENT 46	0	0	1	1	0	0	1	1	1	1
PATIENT 47	0	0	0	1	0	0	0	NA	0	1
PATIENT 48	0	NA	0	NA	0	NA	0	NA	1	NA
PATIENT 49	NA	NA	1	1	0	0	1	2	1	1
PATIENT 50	2	2	0	0	0	0	2	2	NA	NA
PATIENT 51	1	1	NA	0	NA	0	0	0	NA	NA
PATIENT 52	1	1	1	1	0	0	2	1	1	1
PATIENT 53	2	2	1	1	0	0	NA	NA	NA	NA
PATIENT 54	2	1	1	1	0	0	1	2	1	1
PATIENT 55	2	2	1	1	0	0	NA	1	1	1
PATIENT 56	2	0	1	1	1	1	NA	NA	NA	NA
PATIENT 57	2	2	0	0	0	0	0	0	1	1
PATIENT 58	0	0	0	0	0	0	0	0	NA	NA
PATIENT 59	2	2	0	0	0	0	NA	NA	NA	NA
PATIENT 60	1	2	1	1	0	0	1	2	NA	1
PATIENT 61	2	0	0	0	0	0	2	0	1	1
PATIENT 62	0	0	1	1	0	0	0	0	NA	NA
PATIENT 63	2	2	NA	NA	0	0	2	1	1	1
PATIENT 64	1	0	1	1	0	0	NA	NA	NA	NA
PATIENT 65	0	0	0	0	0	0	0	0	NA	NA
PATIENT 66	0	0	0	0	0	0	0	0	NA	NA
PATIENT 67	1	0	NA	0	NA	0	NA	0	NA	NA
PATIENT 68	2	2	1	1	NA	0	NA	2	2	2
PATIENT 69	2	2	1	1	0	0	2	2	NA	NA
PATIENT 70	2		0		0	NA	NA	NA	NA	NA
PATIENT 71	2	1	1	1	0	0	2	NA	NA	NA
PATIENT 72		1	NA	1	NA	0	NA	NA	NA	NA
PATIENT 73	2		0	1	0	NA	NA	NA	2	NA
PATIENT 74	2	2	1	1	1	1	2	NA	2	1
PATIENT 75	1	0	0	0	2	2	2	0	1	1

*NA: Not Available; 0: No radiological findings (healing); 1: Mild radiological findings; 2: Severe radiological findings.

**Fig 3:**
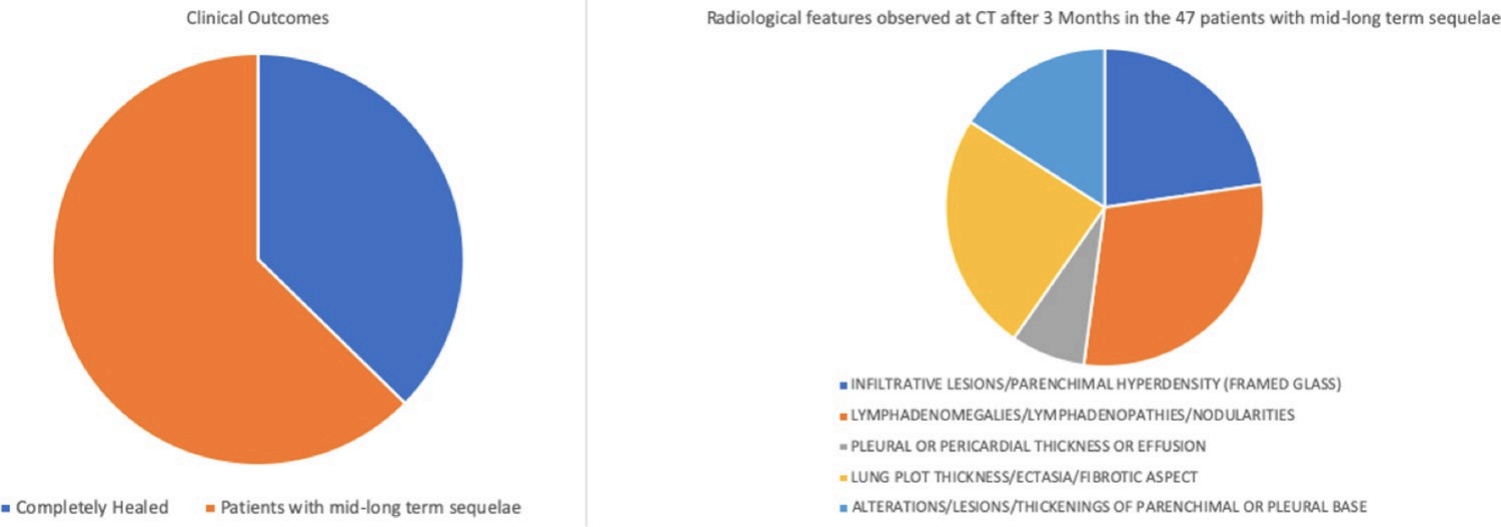
Clinical outcomes. Clinical outcomes (28 patients completely healed + 47 patients showing persistence of radiological sequelae) after 3 months from the hospital admission due to interstitial pneumonia (mild to moderate symptoms) and classification of the radiological sequelae observed in case of disease persistence.

As showed in Fig 3, only 28 of the 75 hospitalized patients (37,3% of the total) were completely healed at month 3 as demonstrated by radiological investigations. Radiological features detected by Computed Tomography after 3 months in the remaining 47 patients (62.7%) are showed in Fig 3 and included the following (each patient could have presented more than one feature): infiltrative lesions or parenchimal hyperdensity with framed glass aspect (detectable in 27 patients; 36% of CT exams), lymphadenomegalies/lymphadenopathies or nodularities (35 patients; 46.6% of CT exams), pleural or pericardial thickness or effusion (9 patients; 12% of CT exams), lung plot thickness/ectasia or fibrotic aspects (29 patients; 38,6% of CT exams), alterations/lesions or thickening of parenchimal or pleural base (observed in 19 patients; 25.3% of CT exams).

Table 3 shows the average values, standard deviation and maximum/minimum values of all the haematochemical parameters dosed in the 75 hospitalized patients. Explorative descriptive analyses have been performed to test the consistency of the baseline characteristics of our hospitalized patients with the results of the evidence published by Kabak et al. [24] confirming that the number of neutrophils and leucocytes were below the normal minimum average count, and also the neutrophil to lymphocyte ratio was suboptimal.

**Table 3:** Average values (with min/max and std deviation) of haematochemical parameters dosed at the time of hospital admission in the 75 hospitalized patients.

Parameter	Average	Standard Deviation	Mínimum Value	Máximum Value
WBC (x 10^3/L)	6,314932	2,196404	3,47	19,08
RBC (x 10^6/L)	4,777808	0,478314	3,36	5,97
HGB (g/dL)	13,40274	1,485576	9,1	16,1
HCT (%)	41,89726	3,91081	30,4	48,5
MCV (fL)	88,08904	7,458693	65	99,6
MCH (pg)	28,1726	2,795048	19,5	32,8
MCHC (g/dL)	31,94795	1,147474	29,8	34,6
PLT(x10^3/æL)	240,4384	64,56607	88	434
RDW-SD (fL)	45,47083	4,963002	34,6	58
RDW-CV (%)	14,2875	1,599466	11,6	20
PDW (fL)	13,64	2,643088	9,2	24,7
MPV (fL)	11,00571	1,071622	8,9	15
P-LCR (%)	33,54638	8,43673	16,5	62
NEUT%	55,56575	7,821697	36,6	71,2
LINF%	33,28356	7,029798	18,7	51,1
MONO%	7,908219	1,758926	4,4	13,9
EOSI%	2,561644	1,365714	0,5	7,3
BASO%	0,653425	0,285327	0,2	1,4
NEUTROF (x 10^3/æL)	3,561096	1,620583	1,33	13,53
LINFOC (x 10^3/æL)	2,064658	0,664099	0,96	4,14
MONOC(x 10^3/L)	0,485694	0,150762	0,21	1,13
EOSIN (x 10^3/L)	0,156027	0,087793	0,03	0,58
BASOF(x 10^3/L)	0,041389	0,022661	0,01	0,12
VES (mm/h)	12,81159	13,34748	2	88
PT (%)	97,33472	9,403343	73,6	123
INR	1,021111	0,059138	0,88	1,15
PTT (s)	32,16364	3,287257	22	41
FIBRINOGEN (mg/dL)	255,9559	58,15585	129	451
D-DIMER (ng/mL)	673,4264	1052,08	91	8188
AZOTEMIA (mg/dL)	37,34	10,23432	20	64
CREATININ (mg/dL)	0,831857	0,158711	0,59	1,25
GOT (U/L)	24,79452	22,7852	12	207
GPT (U/L)	25,39706	17,26885	9	103
GGT (U/L)	30,23611	40,59643	7	337
LDH (U/L)	209,3065	73,01978	146	706
CPK (U/L)	145,3944	453,4975	15	3890
BILIRUBIN TOT (mg/dL)	0,65125	0,309566	0,32	1,95
BILIRUBIN DIRECT (mg/dL)	0,122639	0,059623	0,04	0,33
BILIRUBINA INDIRECT (mg/dL)	0,528889	0,267274	0,27	1,72
PCR (mg/dL)	0,253014	0,321683	0,01	1,63
IL-6 (pg/mL)	42,34648	69,52357	1,5	408

According to the model, despite the small effect depicted, for the parameters showing a statistically significant association with the presence of radiological sequelae at month 3 –namely IL-6 or GPT, platelets and eosinophil count–it was possible to highlight an OR = 0.5 (Table 4), thus meaning that subjects completely healed after 3 months presented half levels of IL-6 at the time of hospitalization (as well as GPT, platelets and eosinophils) compared to patients with mid/long term sequelae.

**Table 4:** Results of the General Linear Model (stepwise full model) used to test for each patient the association between haematochemical examinations performed at the baseline (dependent variables) and the subsequent radiological features at month 3 (independent variable).

Parameter	DF	Estimate	Standard	T vale	Pr(T)	OR
			Error			
***Intercept***	1	-1,35266	0,735325	-1,84	0,0761	
**PLT(x 10^3/æL)**	1	0,002572	0,001031	2,49	0,0186	0,500643
**RDW-SD (fL)**	1	0,031326	0,01323	2,37	0,0248	0,507831
**EOSI%**	1	-0,09729	0,050906	-1,91	0,0659	0,475697
**GPT (U/L)**	1	0,022311	0,005839	3,82	0,0006	0,505578
**Direct BILIRUBIN**	1	-2,28613	1,324487	-1,73	0,095	0,092278
**IL-6 (pg/mL)**	1	-0,00222	0,000832	-2,67	0,0123	0,499444
**Raíz MSE**	0,39906					
**DependentMean**	0,58333					
**R** ^**2**^	0,4722					
**R-SqAdjust**	0,363					
**AIC**	-21,9257					
**AICC**	-16,5923					
**BIC**	-73,9257					
**C(p)**	.					
**PRESS**	6,75673					
**SBC**	-48,841					
**ASE**	0,12829					

*The model kept and run only the parameters showing statistical significance: PLT, RDW-SD, EOSI, GPT, Direct Bilirubin, and IL-6.

## Discussion

Our study involved 75 patients hospitalized in Southern Italy due to symptomatic COVID-19 infection during the months of April and May 2021 (with follow up at 3, 6 and 12 months), which required prolonged oxygen therapy (administered 24 hours per day) along with antinflammatory and antiviral treatments available at the time of the study. On a total of 75 hospitalized patients, only 28 were completely healed after 3 months as documented by negative Computed Tomography (first follow up pneumological visit). The remaining 47 patients showed persistence of radiological sequelae at month 3 that were significantly associated with higher levels of IL-6 measured at the time of hospital admissions, namely two-folds compared to individuals who did not present any radiological alteration at month 3.

These findings seem to be consistent with other literature data and suggest that, among the inflammatory markers, IL-6 could be empirically proposed in medical practice as a possible predictor of mid/long-term unfavorable radiological outcomes. This latter data comes from the use of a Generalized Linear Model (GLM) which considered the baseline haematochemical exams as dependent variables and the 3-month clinical outcome (healing or long-term sequelae based on radiological evidence) as independent variable. Our findings are consistent with those of Herold et al, who found that the levels of IL-6 predict respiratory failure in hospitalized symptomatic COVID-19 patients. Moreover, some authors have already proposed anti-IL-6 as target for the treatment of severe COVID-19 patients [21–23]. We have also performed an explorative descriptive analysis on our haematochemical parameters at the time of hospital admission and we found that the findings of another study carried out by Kabak et al. [24] were confirmed for all the parameters (neutrophil and leucocytes were below the normal minimum average count, and also the neutrophil to lymphocyte ratio was suboptimal) except for lymphocyte and platelet; however it was not possible for us to repeat the comparison with a control group as performed in the study of Kabak et al. [24].

Our data show that COVID-19 patients with moderate to severe symptoms and radiologically confirmed diagnosis of interstitial pneumonia present high levels of IgG, which decline in the subsequent 3–12 months. However, it is known that in addition to specific antibodies response, SARS-COV2 infection is able to trigger additional immunologic response through the activation of T cells (CD4+ T helper lymphocyte memory) potentially conferring to infected subjects a longer protection against the coronavirus that goes beyond the detectable levels of antibodies [25].

Generally speaking, IgG serum levels were always higher than IgM at the moment of hospitalization (75% at T0; n = 12 out of 16 subjects with both data available), after 3 months (72.1%; n = 44 out of 61), after 6 months (56.8%; 25 out of 44), and at one year after hospitalization (60%; 12 out of 20). Interestingly, 18 patients (40.9%) showed IgM levels higher than IgG at month six (T6), and 8 patients (40%) presented the same pattern of IgM being higher than IgG at one year from hospital admission. Overall, IgG serum levels presented a statistically significant decreasing trend from the baseline to month 3 (T3; data available for 66 patients), month 6 (T6; 44 patients with data available for both visits) and month 12 (T12; 14 patients followed up at one year), although the concentration of this kind of antibodies was likely to present variations with small increase between T0 and T3 or T6 in a limited number of patients (n = 6 patients). In all the 14 patients with IgG complete serological data available from T0 to T12 we observed a reduction of IgG after twelve months compared to the baseline. The persistence of specific antibodies IgG after 3 months that was confirmed by our findings is consistent with results of Wajnberg et al. who indicated that this kind of immune response can persist at least for 5 months [8].

IgM serum concentrations showed a statistically significant decreasing trend over the time: IgM were higher at T0 in 78.5% vs. T3 of the 14 patients with both available data; in 89.4% of the 38 subjects tested from T3 to T6; in 83.3% of the 12 serum tests performed from T3 up to T6 and T12. Two patients showed higher levels of IgM at T3 compared to T0, while additional three and two patients increased their IgM from T3 to T6 and from T3 up to T12, respectively. Finally, our findings show the possibility of sporadic increase of IgM after 3 months from the hospitalization, in the frame of negative SARS-COV2 diagnostic tests (PCR ELISA tests performed on throat swabs), as observed in one female patient.

## Conclusions

Based on our findings on 75 patients, COVID-related interstitial pneumonia presenting mild or moderate clinical features (not requiring ICU but only ordinary hospitalization with administration of oxygen as well as pharmacological treatments) might result in mid-term sequelae still detectable at lung Computed Tomography after 3 months from the initial hospital admission. Baseline levels of IL-6 could be proposed as predictor of mid/long term sequelae detectable at imaging at least after 3 months. Individuals infected by SARS-COV2 who develop interstitial pneumonia show early levels of IgG–that are usually higher than IgM–significantly decreasing but still present after 3 and 6 months. Occasionally, it is possible to detect again increases in IgM levels in presence of low levels of IgG and negative PCR ELISA tests for SARS-COV2 RNA.

## Supporting information

S1 File (XLSX)
